# Value of clinical, ultrasonographic and MRI signs as diagnostic differentiators of non-benign lipomatous tumours

**DOI:** 10.1038/s41598-020-77244-2

**Published:** 2020-11-27

**Authors:** Karishma Khan, Elayne Azzopardi, Liberato Camilleri, Ernest A. Azzopardi, Thomas H. Bragg

**Affiliations:** 1 The Welsh Centre for Burns and Plastic Surgery, Heol Maes Eglwys, Swansea, SA66NL UK; 2 Cardiff University Medical School, Heath Park, Cardiff, CF14 4XY UK; 3 University College London, London, UK; 4 University of Malta Tal-Qroqq Campus, Malta GC. EU, UK

**Keywords:** Sarcoma, Signs and symptoms

## Abstract

Suspicion of malignant change within a lipoma is a common and increasing workload within the UK Sarcoma multidisciplinary team (MDT) network, and a source of considerable patient anxiety. Currently, there is no lipoma-specific data, with regard to which clinical or radiographic features predict non-benign histology, or calculate an odds-ratio specific to a lipomatous lesion being non-benign. We performed a 9-year, double-blind, unmatched cohort study, comparing post-operative histology outcomes (benign versus non-benign) versus 15 signs across three domains: Clinical (size of tumour, depth, growth noticed by patient, previous lipoma, patient felt pain), Ultrasonographic (size, depth, vascularity, heterogenous features, septae) and MRI (size, depth, vascularity, heterogenous features, septae, complete fat signal suppression). Receiver operating characteristic (ROC) analysis, odds ratios and binary logistic regression analysis was performed double-blind. When each sign is considered independently, (ROC analysis, followed by binary logistic regression) only Ultrasound depth is a significant predictor (p = 0.044) of a histologically non-benign lipoma. Ultrasonographically determined vascularity and septation were not statistically significant predictors. None of the clinical signs were statistically significant (p > 0.05). Of the MRI signs none was statistically significant (p > 0.05). However, heterogeneous MRI features fared better than MRI depth. Ultrasound signs (Pseudo R-Square = 0.105) are more predictive of the post-operation histology outcome than Clinical signs (Pseudo R-Square = 0.082) or MRI tests (Pseudo R-Square = 0.052) Ultrasound and Clinical tests combined (Pseudo R-Square = 0.147) are more predictive of the post-operation histology outcome than MRI tests (Pseudo R-Square = 0.052). This work challenges the traditional perception of “red-flag” signs when applied to lipomatous tumours. We provide accurate data upon which an informed choice can be made, and provides a robust bases for expedited risk/benefit. The importance of an experienced and cohesive MDT network is emphasised.

## Introduction

Tumors of adipose represent both the commonest (50%) of all benign mesenchymal tumours as lipomas, and the commonest group of malignant mesenchymal neoplasms (liposarcomas), which incurs a considerable disease burden and healthcare cost^1,2^. It is therefore surprising that there is no literature that explores the value of clinical, ultrasonoraphic, and magnetic resonance imaging signs in current clinical use, alone or in combination, specifically in differentiating benign from non-benign adipose tumours.

The UK National Institute for Health and Care Excellence (NICE) recognises the difficulty in distinguishing benign from malignant tumours as the principal problem in their management^3^. Discerning benign from malginant is the key decision that impacts on morbidity, mortality, and service delivery^4^. Besides ultrasonograpy and magnetic resonance imaging, current consensus opinion both by NICE and the British Sarcoma Group advocates any lump with any size, or size > 5 cm, or deep to the deep fascia, or painful is considered higher risk for potential malignancy until proven otherwise, as a blanket approach to any soft tissue sarcoma^5,6^.

### Study aims

To determine the diagnostic value of 15 signs across three domains, as used in standard sarcoma multidisciplinary team (MDT) practice, for primary tumours of adipose: Clinical (size of tumour, depth, growth noticed by patient, previous lipoma, new onset of pain), Ultrasonographic (size, depth, vascularity, heterogenous features septae) and MRI (size, depth, vascularity, heterogenous features, septae, complete fat signal suppression).

#### Hypothesis

That current clinical, ultrasonographic and magnetic resonance signs do not equally predict and differentiate benign from non-benign lipomatous tumours.

### Operational definitions

We adopted A pragmatic, benign versus non-benign, binary approach, in keeping with World Health Organisatiion (WHO) defnitions^7^.

*Gold standard* was determined as the post-operative laboratory report histopathology, subject to standard UK National Health Service quality control criteria, and reported by an experienced validated MDT histopathologist.

“Benign” was defined as a lipoma, confirmed as benign on histologic report. “Non-benign” was determined as liposarcoma, including atypical lipomatous tumour/well-differentiated liposarcoma (which includes the adipocytic, sclerosing, inflammatory and spindle cell variants); de-differentiated liposarcoma; myxoid liposarcoma; and pleomorphic liposarcoma^7^.

Red flag Signs: The classical red flag signs were taken as defined by the National Institute for Health and Care Excellence (3, 5). These are reproduced in scheme 1.

#### Scheme 1

Features suggestive of malignancy in a lump.

- Lump > 5 cm
- Lump increasing in size
- Lump deep to the fascia
- Pain.

### Inclusion criteria

Patients with both histology, ultrasonographic or magnetic imaging reporting, presenting with suspected non-syndromic tumours of adipose, operated within the Wales Sarcoma Service between 2010 and March 2018.

### Blinding

Data was anonymised at source. Data coding and primary analysis was performed blind. Statistical analysis was performed blind by an independent bio-statistician.

### Ethics

This study was performed using retrospective anlysis of audit data anonymised at source. This was confirmed using the UK Health Research Authority’s online decisional analysis tool, and seconded by institutional board correspondence^8,9^. All analysis methods were carried out in accordance with governing institutional guidelines and regulations at Swansea University and affiliated hospitals^10^ (clinical audit/service evaluation data).

## Methods

We performed a 9-year, single sarcoma network, double-blind, unmatched cohort study, comparing post-operative histology outcomes (benign versus non-benign) versus 15 signs across three domains: Clinical (size of tumour, depth, growth noticed by patient, previous lipoma, patient felt pain), Ultrasonographic (size, depth, vascularity, heterogenous features septae) and MRI (size, depth, vascularity, heterogenous features, septae, complete fat signal suppression). The data was collected from the Welsh Sarcoma Service and incorporates the work of a senior, validated MDT team working according to British Sarcoma Standards. Data coding was performed as per Supplementary material^1^.

For each parameter, sensitivity, specificity, positive predictive value, negative predictive value, was followed by Reported-Observer Curve (ROC) analysis. Binary Logistic Regression Analysis was then used to investigate the collective contribution of signs in each domain area. Odds ratios were then calculated. Statistical significance was considered at p < 0.05. Data analysis was performed with SpSS for Windows (IBM Corp. Released 2017. Version 25.0. Armonk, NY: IBM Corp).

## Results

**Figure 1:**
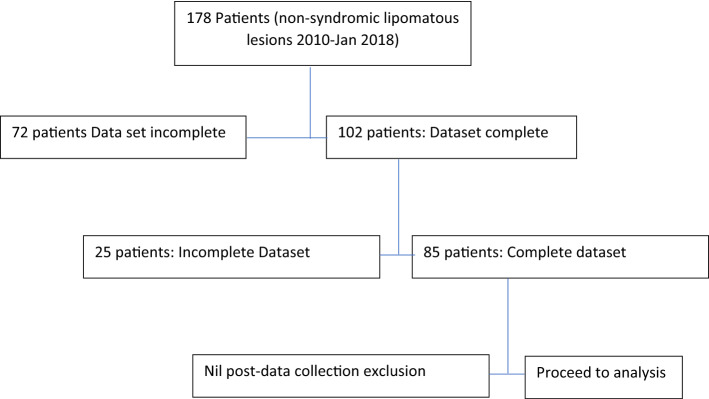
Attrition diagram to suppport exlusion of patients.

Of 178 patients originally fulfilling inclusion criteria, histopathology reports were available in 106. Of these 25 lacked either ultrasonographic or magnetic resonance imaging data and were excluded, and the remainder (81) were included in the study (Fig. 1). To our knowledge this is the largest reported cohort of patients in the Literature to date.

For each domain, sensitivity, specificity, positive predictive value, and negative predictive value are reported in Table 1.

**Table 1:** Sensitivty, specificity, positive and negative predictive value for each parameter individually.

Parameter	Sens (%)	Spec (%)	Ppv (%)	Npv (%)
**Clinical**				
Size > 5 cm	92.3	19.1	17.9	92.8
Depth	53.85	66.18	23.33	88.24
Growth	38.46	39.71	10.87	77.14
Pain	23.08	73.53	14.29	83.33
Previous lipoma	15.38	91.18	25.00	84.93
**Ultrasonographic**				
Size > 5 cm	61.54	31.34	14.81	80.77
Depth	46.15	75.76	27.27	87.72
Vascularity	33.33	77.61	21.05	86.67
Heterog. features	7.69	92.54	16.67	83.73
Septation	15.38	94.03	33.33	85.14
**MRI**				
Size > 5 cm	66.67	9.76	13.95	57.14
Depth	55.56	56.10	21.74	85.19
Vascularity	0	85.36	0	79.55
Heterog features	33.33	82.93	30.00	v85.00

Sens: sensitivity; spec: specificty; PpV: Postive predictive value; NpV: negative predictive value; heterog: heterogenous.

### Clinical domain

#### Size of lipoma

ROC analysis shows this has some predictive power. However, p-value (0.516) exceeds the 0.05 level of significance. This is mainly attributed to the fact that the number of false positives (55) is quite large.

#### Depth of lipoma

The area under the ROC curve (0.600) exceeds the area under the 45-degree line (0.5) indicating that the depth of tumour has some predictive power. However, the p-value (0.255) exceeds the 0.05 level of significance indicating that this area is not significantly larger than 0.5. This is mainly attributed to the fact that the number of false positives (23) is quite large.

#### Growth noted by patient

The area under the ROC curve (0.391) is less than the area under the 45-degree line (0.5) indicating that growth noticed by patients has no predictive power. This is mainly attributed to the fact that the number of false negatives (8) and false positives (41) are larger than the true positives (5) and true negatives (27).

#### Previous lipoma

The area under the ROC curve (0.533) exceeds the area under the 45-degree line (0.5) indicating that previous lipoma has some predictive power. However, the p-value (0.709) exceeds the 0.05 level of significance indicating that this area is not significantly larger than 0.5. This is mainly attributed to the fact that the number of false negatives (11) is quite large.

#### Pain

The area under the ROC curve (0.483) is less than the area under the 45-degree line (0.5) indicating that the pain felt by patients has no predictive power. This is mainly attributed to the fact that the number of false negatives (10) is larger than the number of true positives (3).

Although none of three predictors are significant, depth of tumour is the better of the three, followed by size of tumour and previous Lipoma. When the above three parameters we investigated collectively as potential predictors using a binary logistic regression model, the parameters explain only 8.2% of the post-operation histology outcome. The Odds ratios are reported in Table 2.

**Table 2:** Odds of a non-benign histology for each clinical sign evaluated.

Parameter	Odds ratio
lump > 5 cm	If size of tumour is 5 cm or more, the odds that post-operation histology yields a non-benign tumour is 3.633 times when size of tumour is less than 5 cm
Tethered to fascia or deeper structures	If the tumour includes fascia or is deep to fascia, the odds that post-operation histology yields a non-benign tumour is 2.257 times when tumour depth is superficial
Previous lipoma	If previous lipoma is in the same position, the odds that post-operation histology yields a non-benign tumour is 3.341 times when no previous lipomas are reported

### Ultrasound domains

Ultrasonographic size: The area under the ROC curve (0.464) is less than the area under the 45-degree line (0.5) indicating that size of ultrasound has no predictive power. This is mainly attributed to the fact that the number of false positives (46) is large.

Ultrasonographic depth: The area under the ROC curve (0.610) exceeds the area under the 45-degree line (0.5) indicating that tumours occuring adjacent to or deep to fascia has some predictive power. However, the p-value (0.214) exceeds the 0.05 level of significance indicating that this area is not significantly larger than 0.5. This is mainly attributed to the fact that the number of false positives (16) is quite large.

Ultrasonographic evidence of tumour vascularity: the area under the ROC curve (0.555) exceeds the area under the 45-degree line (0.5) indicating that the ultrasound vascularity has some predictive power. However, the p-value (0.548) exceeds the 0.05 level of significance indicating that this area is not significantly larger than 0.5. This is mainly attributed to the fact that the number of false positives (15) is quite large.

Ultrasonographic evidence of tumour heterogeneity: The area under the ROC curve (0.501) exceeds the area under the 45-degree line (0.5) by a very small margin indicating that the ultrasound heterogeneous feature has very little predictive power. Moreover, the p-value (0.990) exceeds the 0.05 level of significance indicating that this area is not significantly larger than 0.5. This is mainly attributed to the fact that the number of false negatives (12) is quite large.

Ultrasound detection of septae: The area under the ROC curve (0.547) exceeds the area under the 45-degree line (0.5) indicating that the ultrasound detected septae has some predictive power. However, the p-value (0.593) exceeds the 0.05 level of significance indicating that this area is not significantly larger than 0.5. This is mainly attributed to the fact that the number of false negatives (11) is quite large.

### Binary logistic regression of ultrasonographic domains

Logistic regression analysis was used to investigate the collective contribution of Ultrasound depth, ultrasound detected vascularity and ultrasound septae in predicting the outcome (non-benign, benign) of the post-operation histology. This three-predictor logistic regression model explains 10.5% of the post-operation histology outcome (Nagelkerke Pseudo R-Square = 0.105). Moreover, ultrasound tumour depth is a significant predictor (p = 0.044). Consequently it appears that ultrasound depth is the better of the three, followed by ultrasonographic evidence of vascularity and tumour septation.

The odds ratios, displayed in Table 3, all indicate that tumours of size 5 cm or more, tumours which abuts fascia or are deep to fascia and previous lipoma in the same position increase the risk that the post-operation histology yields a non-benign tumour since these odds are all larger than 1. However, these odds ratios are not significantly larger than 1 because the p-values exceed the 0.05 level of significance.

**Table 3:** Odds of a non-benign histology for each clinical sign evaluated.

Parameter	Odds ratio
Ultrasound tumour depth	If ultrasound tumour depth is fascial or sub-fascial, the odds that post-operation histology yields a non-benign tumour is 4.059 times when ultrasound depth is superficial
Vascularity	If the tumour displays vascularity, the odds that post-operation histology yields a non-benign tumour is 2.471 times when tumour does not invade microvasculature
Septation	If ultrasound detected septae are present, the odds that post-operation histology yields a non-benign tumour is 1.394 times when septae are not present

### MRI results

Size: The area under the ROC curve (0.382) is less than the area under the 45-degree line (0.5) indicating that MRI size has no predictive power. This is mainly attributed to the fact that the number of false positives (37) is large.

Depth: The area under the ROC curve (0.558) exceeds the area under the 45-degree line (0.5) indicating that the MRI depth has some predictive power. However, the p-value (0.587) exceeds the 0.05 level of significance indicating that this area is not significantly larger than 0.5. This is mainly attributed to the fact that the number of false positives (18) is quite large.

MRI detected vascularity: The area under the ROC curve (0.427) is less than the area under the 45-degree line (0.5) indicating that MRI detected tumour vascularity has no predictive power. This is mainly attributed to the fact that the number of false negatives (9) is large.

MRI heterogenous features: The area under the ROC curve (0.581) exceeds the area under the 45-degree line (0.5) indicating that the MRI evidence of heterogeneous features have some predictive power. However, the p-value (0.449) exceeds the 0.05 level of significance indicating that this area is not significantly larger than 0.5). This is mainly attributed to the fact that the number of false negatives (6) is quite large.

MRI Septae: The area under the ROC curve (0.409) is less than the area under the 45-degree line (0.5) indicating that MRI evidence of septation has no predictive power. This is mainly attributed to the fact that the number of false negatives (8) is large.

MRI fat suppression: The area under the ROC curve (0.336) is less than the area under the 45-degree line (0.5) indicating that Fat completely suppressed has no predictive power. This is mainly attributed to the fact that the number of false negatives (8) and false positives (18) are large.

Logistic regression for MRI criteria. Logistic regression analysis was used to investigate the collective contribution of MRI depth and MRI heterogeneous features in predicting the outcome (non-benign, benign) of the post-operation histology. This two-predictor logistic regression model explains 5.2% of the post-operation histology outcome (Nagelkerke Pseudo R-Square = 0.052). Although none of these two predictors are significant, MRI heterogeneous features is the better of the two, followed by MRI depth.

Table 4 displays the odds ratios. The odds ratios all indicate that tumours that involve fascia or invades to fascia on MRI and heterogeneous features on MRI increase the risk that the post-operation histology yields a non-benign tumour since these odds are all larger than 1. However, these odds ratios are not significantly larger than 1 because the p-values exceed the 0.05 level of significance.

**Table 4:** Odds of a non-benign histology for each clinical sign evaluated.

Parameter	Odds ratio
MRI depth	If MRI depth involves fascia or reaches deep to fascia, the odds that post-operation histology yields a non-benign tumour is 1.708 times when MRI depth is superficial to fascia
MRI features are heterogeneous	If MRI features are heterogeneous, the odds that post-operation histology yields a non-benign tumour is 2.557 times when MRI features are homogeneous

Additionally, we used logistic regression analysis was used to investigate the collective contribution of size of tumour, depth of tumour, previous Lipoma, ultrasound depth, ultrasound tumour vascularity and ultrasound septae in predicting the outcome (non-benign, benign) of the post-operation histology. Now, this six-predictor logistic regression model now explains 14.7% of the post-operation histology outcome (Nagelkerke Pseudo R-Square = 0.147).

## Result summary

**Table:** 

Of the clinical tests, depth of tumour is the best predictor of the post-operation histology outcome (non-benign, benign), followed by size of tumour and previous lipoma
Of the ultrasound tests, ultrasound depth is the best predictor of the post-operation histology outcome (non-benign, benign), followed by ultrasound tumour vascularity and ultrasound septae. Depth is also statistically significant on ROC analysis
Of the MRI tests, heterogeneous features on MRI is the best predictor of the post-operation histology outcome (non-benign, benign), followed by MRI depth
Of the clinical and ultrasound tests combined, ultrasound depth is the best predictor of the post-operation histology outcome (non-benign, benign), followed by size of tumour, ultrasound vascularity and previous lipoma
Ultrasound tests (Pseudo R-Square = 0.105) are more predictive of the post-operation histology outcome than Clinical tests (Pseudo R-Square = 0.082) and MRI tests (Pseudo R-Square = 0.052)
Ultrasound and Clinical tests combined (Pseudo R-Square = 0.147) are more predictive of the post-operation histology outcome than MRI tests (Pseudo R-Square = 0.052)

## Discussion

The decision when to operate can be difficult with regard to tumours of adipose. An important aim in sarcoma management is early diagnosis and prompt referral^11^. Our study shows that the classical “red-flag” signs^5^ that are associated with malignant change are of limited value in the differentiation of lipomatous tumours. Much of the bases for the current “red flag symptoms” is based on the work of Johnson Pysent and Grimer^12^. These authors used a weighting scheme to measure the likelihood of a malignant lesion and then converted this weight to a probability using the logistic function, where larger weights corresponded to higher probabilities of malignant lesions. Our work differs in serveral ways. Firstly, our approach is model-based where the contribution of each parameter is investigated individually using ROC curve analyses and then collectively with other parameters using Binary Logistic models. These models, which are appropriate for analyzing binary responses, yield odds ratios which measure the likelihood of non-benign histology in the presence of a parameter when compared to its absence. Moreover, we only included lipomatous tumours and data retrieval was double cross-checked. Statistical analysis was performed blind by an experienced statistician.

Only ultrasonographically determined tumour depth withstood binary logistic regression analysis to emerge as a significant predictor of the post-operative histology. Further, our results show that combined ultrasonographic and clinical examination, may be more predictive of post-operative histology when compared to magnetic resonance imaging in isolation. These results may also have implications on service delivery, access and co-production decision sharing. Patient anxiety and reduced quality of life often accompany the interim period between referral and final diagnosis. The odds ratios presented herein for each sign, contribute to informing decisions taken jointly between patient and the clinical team, and clarifying informed consent. In the presence of conflicting clinical, or radiological evidenced, these findings may facilitate MDT co-production decision making based upon the predictive strength of the individual signs within each domain. Further, the predictive superiority of combined clinical plus ultrasonographic examination, in particularly depth on ultrasound, have implications for service delivery and access. The Welsh Sarcoma Service now offers combined sarcoma screening clinics which are both rapid access and cost-saving compared to MRI imaging. Whilst the latter retains an important role in diagnostics, its use may be more focused on surgical planning and possible morbidity from surgery.

Limitations and direction of future research: Our findings are dependent on a caveat of a highly experienced sarcoma MDT being inolved in clinical examination. We also noted that some of the main effects (ultrasound depth, size of tumour, ultrasound vascularity and previous lipoma) yielded p-values that exceeded the 0.05 level of significance by a small margin. Even though, to our knowledge, this was the study with the largest study sample size reported in the literature to date, we cannot exlcude the possibility that these predictors could be significant if the sample size had to be increased further. It is known that when conducting hypothesis testing, the p-value depends heavily on the sample size and it is very unlikely to attain statistical significance in the presence of heterogeneous responses.

These findings and their implications have been incorporated in the Welsh Sarcoma Service Multidisicplinary Meeting policy and form the bases our current practice.

## Supplementary information

Supplementary information.
