# Supplementary material for: Value of clinical, ultrasonographic and MRI signs as diagnostic differentiators of non-benign lipomatous tumours
Source: Sci Rep. 2020 Nov 27;10:20756. doi: 10.1038/s41598-020-77244-2 (PMC7695823; doi:10.1038/s41598-020-77244-2)
Supplement: Supplementary file 1 — Supplementary information. [file 41598_2020_77244_MOESM1_ESM.docx]

**Supplementary Material 1: data coding**

**Dependent variable**

Post-operation histology  1=non-benign, 2 = benign

**Predictors (Independent variables)**

size of tumour

1 = non benign (clinical size 5 cm or more)

2= benign (size less than 5 cm)

depth

1= non-benign (includes fascia or deep to fascia)

2= benign (superficial)

growth noticed by patient

1=Nonbenign (any recent growth)

2= benign (no growth)

previous lipoma

1= previous lipoma in the same position

2= patient does not report any previous lipomas

size on ultrasound

1 = non benign (ultrasoundl size 5 cm or more)

2= benign (ultrasound size less than 5 cm)

us.depth

1= non benign ( fascial or sobfascial)

2= benign (superficial)

us.vascular invasion

1= non-benign (invades microvasculature)

2= benign (does not invade microvasculature)

us.heterogenous features

1= non-benign (morphologic heterogeneity)

2= benign (no morphologic heterogeneity)

us.septae

1= non-benign (septae present)

2= benign (no septae)

mri.size

1= non-benign (5 cm or more on mri)

2= benign (less than 5 cm on mri)

mri.depth

1= non-benign (involves fascia or invades deep to fascia on mRI)

2= benign (superficial to fascia on MRI)

mri.vascular invasion

1= non-benign (invades microvasculature on mRI)

2= benign (does not invade microvasculature MRI)

mri.heteregenous features

1= non-benign (heterogenous)

2= benign (homogenous)

mri.septae

1= non-benign (septae present)

2= benign (septae absent)

fat completely suppressed on mri

1= non-benign (fat not completely suppressed)

2= benign (fat completely suppressed)

**Supplementary Material 2: Complete statistical work-up with lay summary**

**Size of tumour**

|  | | Post-operation histology | | Total |
| --- | --- | --- | --- | --- |
|  |  | Non-Benign | Benign |  |
| Size of tumour | Clinical size 5cm or more (Non-benign) | 12 | 55 | 67 |
|  | Clinical size less than 5cm (Benign) | 1 | 13 | 14 |
| Total | | 13 | 68 | 81 |


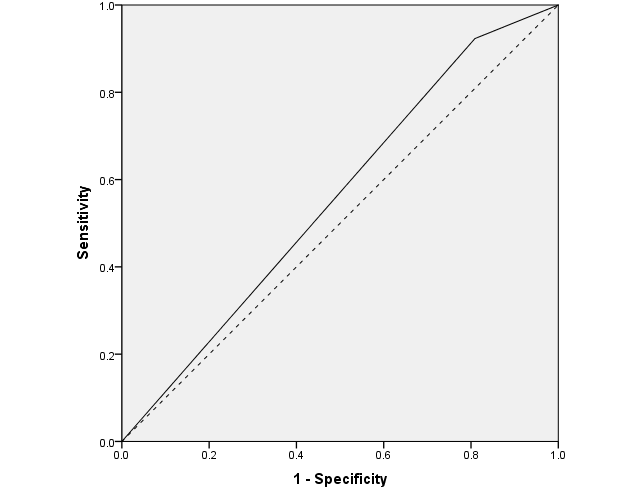


Sensitivity = 12 / 13 = 0.9231 = 92.31%

Specificity = 13 / 68 = 0.1912 = 19.12%

Positive Predictive Value = 12 / 67 = 0.1791 = 17.91%

Negative Predictive Value = 13 / 14 = 0.9286 = 92.86%

| Area Under the Curve (Size of tumour) | | | | |
| --- | --- | --- | --- | --- |
| Area | Std. Error | P-value | 95% Confidence Interval | |
|  |  |  | Lower Bound | Upper Bound |
| 0.557 | 0.082 | 0.516 | 0.396 | 0.718 |

The area under the ROC curve (0.557) exceeds the area under the 45-degree line (0.5) indicating that the size of tumour has some predictive power. However, p-value (0.516) exceeds the 0.05 level of significance indicating that this area is not significantly larger than 0.5. This is mainly attributed to the fact that the number of false positives (55) is quite large.

**Depth of tumour**

|  | | Post-operation histology | | Total |
| --- | --- | --- | --- | --- |
|  |  | Non-Benign | Benign |  |
| Depth | Includes fascia or deep to fascia (Non-benign) | 7 | 23 | 30 |
|  | Superficial (Benign) | 6 | 45 | 51 |
| Total | | 13 | 68 | 81 |


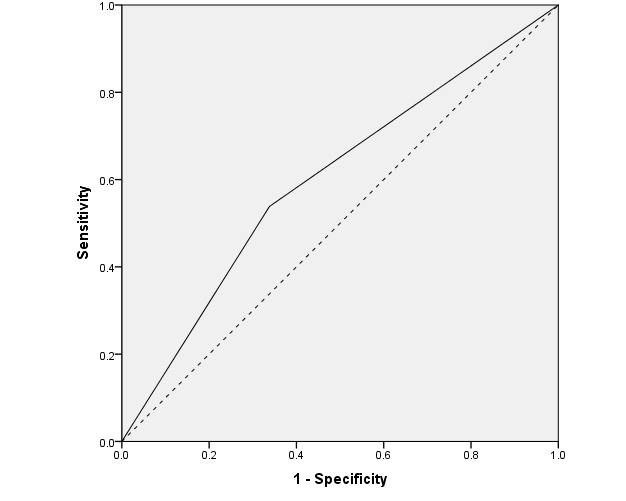


Sensitivity = 7 / 13 = 0.5385 = 53.85%

Specificity = 45 / 68 = 0.6618 = 66.18%

Positive Predictive Value = 7 / 30 = 0.2333 = 23.33%

Negative Predictive Value = 45 / 51 = 0.8824 = 88.24%

| Area Under the Curve (Depth of tumour) | | | | |
| --- | --- | --- | --- | --- |
| Area | Std. Error | P-value | 95% Confidence Interval | |
|  |  |  | Lower Bound | Upper Bound |
| 0.600 | 0.088 | 0.255 | 0.428 | 0.772 |

The area under the ROC curve (0.600) exceeds the area under the 45-degree line (0.5) indicating that the depth of tumour has some predictive power. However, the p-value (0.255) exceeds the 0.05 level of significance indicating that this area is not significantly larger than 0.5. This is mainly attributed to the fact that the number of false positives (23) is quite large.

**Growth noticed by patient**

|  | | Post-operation histology | | Total |
| --- | --- | --- | --- | --- |
|  |  | Non-Benign | Benign |  |
| Growth noticed by patient | Any recent growth (Non-benign) | 5 | 41 | 46 |
|  | No growth (Benign) | 8 | 27 | 35 |
| Total | | 13 | 68 | 81 |


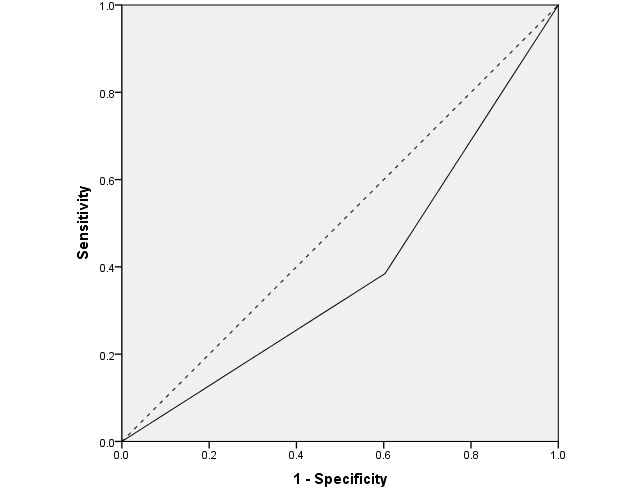


Sensitivity = 5 / 13 = 0.3846 = 38.46%

Specificity = 27 / 68 = 0.3971 = 39.71%

Positive Predictive Value = 5 / 46 = 0.1087 = 10.87%

Negative Predictive Value = 27 / 35 = 0.7714 = 77.14%

| Area Under the Curve (Growth noticed by patient) | | | | |
| --- | --- | --- | --- | --- |
| Area | Std. Error | P-value | 95% Confidence Interval | |
|  |  |  | Lower Bound | Upper Bound |
| 0.391 | 0.086 | 0.214 | 0.223 | 0.559 |

The area under the ROC curve (0.391) is less than the area under the 45-degree line (0.5) indicating that growth noticed by patients has no predictive power. This is mainly attributed to the fact that the number of false negatives (8) and false positives (41) are larger than the true positives (5) and true negatives (27).

**Previous Lipoma**

|  | | Post-operation histology | | Total |
| --- | --- | --- | --- | --- |
|  |  | Non-Benign | Benign |  |
| Previous lipoma | Previous lipoma in the same position (Non-benign) | 2 | 6 | 8 |
|  | No report of previous lipoma (Benign) | 11 | 62 | 73 |
| Total | | 13 | 68 | 81 |


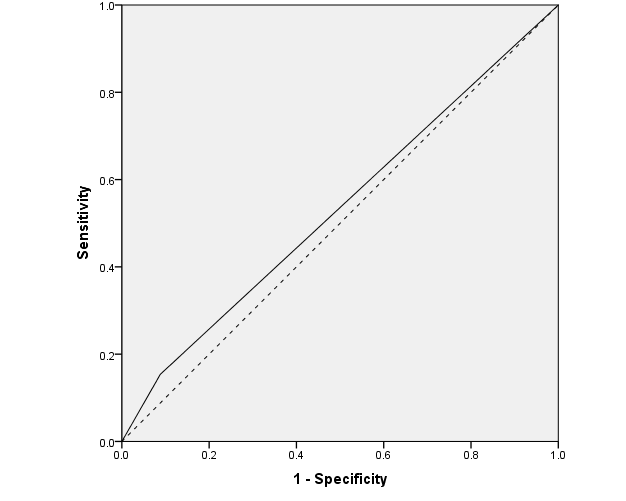


Sensitivity = 2 / 13 = 0.1538 = 15.38%

Specificity = 62 / 68 = 0.9118 = 91.18 %

Positive Predictive Value = 2 / 8 = 0.2500 = 25.00 %

Negative Predictive Value = 62 / 73 = 0.8493 = 84.93 %

| Area Under the Curve (Previous Lipoma) | | | | |
| --- | --- | --- | --- | --- |
| Area | Std. Error | P-value | 95% Confidence Interval | |
|  |  |  | Lower Bound | Upper Bound |
| 0.533 | 0.091 | 0.709 | 0.355 | 0.710 |

The area under the ROC curve (0.533) exceeds the area under the 45-degree line (0.5) indicating that previous lipoma has some predictive power. However, the p-value (0.709) exceeds the 0.05 level of significance indicating that this area is not significantly larger than 0.5. This is mainly attributed to the fact that the number of false negatives (11) is quite large.

**Patient felt pain**

|  | | Post-operation histology | | Total |
| --- | --- | --- | --- | --- |
|  |  | Non-Benign | Benign |  |
| Patient felt pain | Yes (Non-Benign) | 3 | 18 | 21 |
|  | No (Benign) | 10 | 50 | 60 |
| Total | | 13 | 68 | 81 |


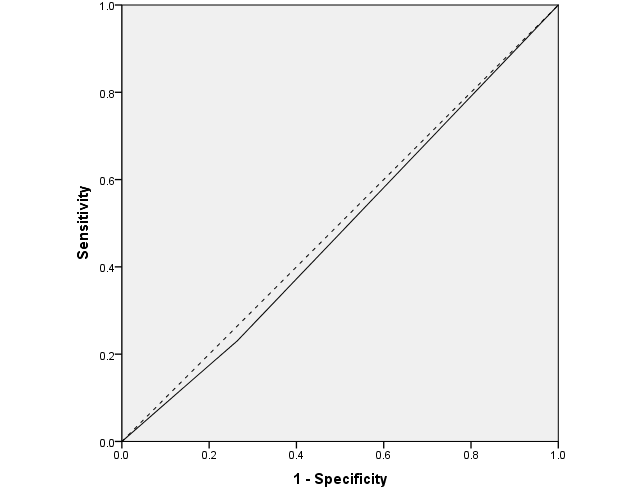


Sensitivity = 3 / 13 = 0.2308 = 23.08%

Specificity = 50 / 68 = 0.7353 = 73.53%

Positive Predictive Value = 3 / 21 = 0.1429 = 14.29%

Negative Predictive Value = 50 / 60 = 0.8333 = 83.33 %

| Area Under the Curve (Patient felt pain) | | | | |
| --- | --- | --- | --- | --- |
| Area | Std. Error | P-value | 95% Confidence Interval | |
|  |  |  | Lower Bound | Upper Bound |
| 0.483 | 0.087 | 0.847 | 0.313 | 0.653 |

The area under the ROC curve (0.483) is less than the area under the 45-degree line (0.5) indicating that the pain felt by patients has no predictive power. This is mainly attributed to the fact that the number of false negatives (10) is larger than the true positives (3).

**Binary Logistic Regression Analysis (Clinical Tests)**

Logistic regression analysis was used to investigate the collective contribution of size of tumour, depth of tumour and previous Lipoma in predicting the outcome (non-benign, benign) of the post-operation histology. The rationale of using Binary Logistic regression analysis is that the dependent variable (post-operation histology) is a categorical variable with two possible outcomes (non-benign, benign). (Nagelkerke Pseudo R-Square = 0.082). Although none of three predictors are significant, depth of tumour is the better of the three, followed by size of tumour and previous Lipoma.

The second table displays the odds ratios. The odds ratios all indicate that tumours of size 5cm or more, tumours which include fascia or deep to fascia and previous lipoma in the same position increase the risk that the post-operation histology yields a non-benign tumour since these odds are all larger than 1. However, these odds ratios are not significantly larger than 1 because the p-values exceed the 0.05 level of significance.

| Effect | Model Fitting Criteria | Likelihood Ratio Tests | | |
| --- | --- | --- | --- | --- |
|  | -2 Log Likelihood of Reduced Model | Chi-Square | df | P-value |
| Intercept | 15.857 | 0.000 | 0 | . |
| Size of tumour | 17.356 | 1.499 | 1 | 0.221 |
| Depth of tumour | 17.543 | 1.686 | 1 | 0.194 |
| Previous lipoma | 17.214 | 1.357 | 1 | 0.244 |

| Parameter Estimates | | | | | | | |
| --- | --- | --- | --- | --- | --- | --- | --- |
| Post-operation histology | | B | Std. Error | Wald | df | P-value | Odds ratio |
|  | Intercept | -3.283 | 1.214 | 7.315 | 1 | 0.007 |  |
|  | Size = 5cm or more | 1.290 | 1.187 | 1.180 | 1 | 0.277 | 3.633 |
|  | Size = Less than 5cm | 0 | . | . | 0 | . | . |
|  | Depth = Fascia/Deep to fascia | 0.814 | 0.629 | 1.675 | 1 | 0.196 | 2.257 |
|  | Depth = Superficial | 0 | . | . | 0 | . | . |
|  | Previous lipoma = Same position | 1.206 | 0.992 | 1.478 | 1 | 0.224 | 3.341 |
|  | Previous lipoma = No lipomas | 0 | . | . | 0 | . | . |

If size of tumour is 5cm or more, the odds that post-operation histology yields a non-benign tumour is 3.633 times when size of tumour is less than 5cm.

If the tumour includes fascia or is deep to fascia, the odds that post-operation histology yields a non-benign tumour is 2.257 times when tumour depth is superficial.

If previous lipoma is in the same position, the odds that post-operation histology yields a non-benign tumour is 3.341 times when no previous lipomas are reported.

**Size of Ultrasound**

|  | | Post-operation histology | | Total |
| --- | --- | --- | --- | --- |
|  |  | Non-Benign | Benign |  |
| Size of Ultrasound | Ultrasound size 5cm or more (Non-Benign) | 8 | 46 | 54 |
|  | Ultrasound size less than 5cm (Benign) | 5 | 21 | 26 |
| Total | | 13 | 67 | 80 |


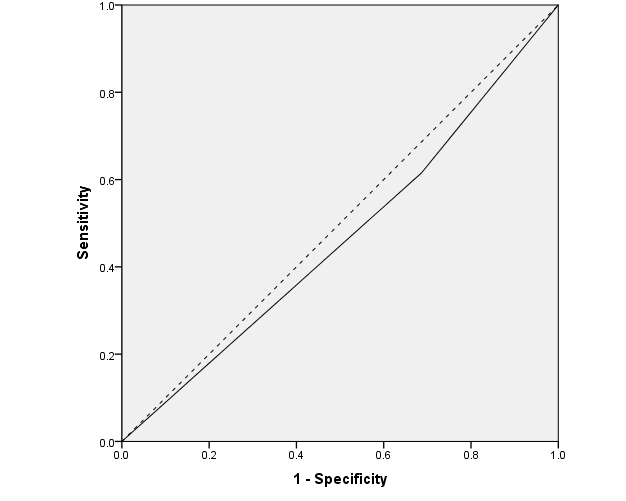


Sensitivity = 8 / 13 = 0.6154 = 61.54%

Specificity = 21 / 67 = 0.3134 = 31.34%

Positive Predictive Value = 8 / 54 = 0.1481 = 14.81%

Negative Predictive Value = 21 / 26 = 0.8077 = 80.77%

| Area Under the Curve (Size of ultrasound) | | | | |
| --- | --- | --- | --- | --- |
| Area | Std. Error | P-value | 95% Confidence Interval | |
|  |  |  | Lower Bound | Upper Bound |
| 0.464 | 0.089 | 0.686 | 0.290 | 0.639 |

The area under the ROC curve (0.464) is less than the area under the 45-degree line (0.5) indicating that size of ultrasound has no predictive power. This is mainly attributed to the fact that the number of false positives (46) is large.

**Ultrasound Depth**

|  | | Post-operation histology | | Total |
| --- | --- | --- | --- | --- |
|  |  | Non-Benign | Benign |  |
| Ultrasound Depth | Fascial or sub fascial (Non-Benign) | 6 | 16 | 22 |
|  | Superficial (Benign) | 7 | 50 | 57 |
| Total | | 13 | 66 | 79 |


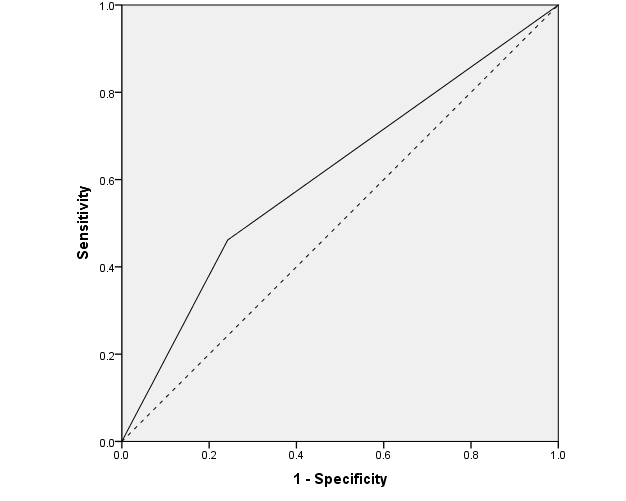


Sensitivity = 6 / 13 = 0.4615 = 46.15%

Specificity = 50 / 66 = 0.7576 = 75.76%

Positive Predictive Value = 6 / 22 = 0.2727 = 27.27%

Negative Predictive Value = 50 / 57 = 0.8772 = 87.72%

| Area Under the Curve (Depth of ultrasound) | | | | |
| --- | --- | --- | --- | --- |
| Area | Std. Error | P-value | 95% Confidence Interval | |
|  |  |  | Lower Bound | Upper Bound |
| 0.610 | 0.090 | 0.214 | 0.434 | 0.786 |

The area under the ROC curve (0.610) exceeds the area under the 45-degree line (0.5) indicating that the depth of tumour has some predictive power. However, the p-value (0.214) exceeds the 0.05 level of significance indicating that this area is not significantly larger than 0.5. This is mainly attributed to the fact that the number of false positives (16) is quite large.

**Ultrasound Vascular Invasion**

|  | | Post-operation histology | | Total |
| --- | --- | --- | --- | --- |
|  |  | Non-Benign | Benign |  |
| Ultrasound vascular invasion | Invades microvasculature (Non-Benign) | 4 | 15 | 19 |
|  | Does not invade microvasculature (Benign) | 8 | 52 | 60 |
| Total | | 12 | 67 | 79 |


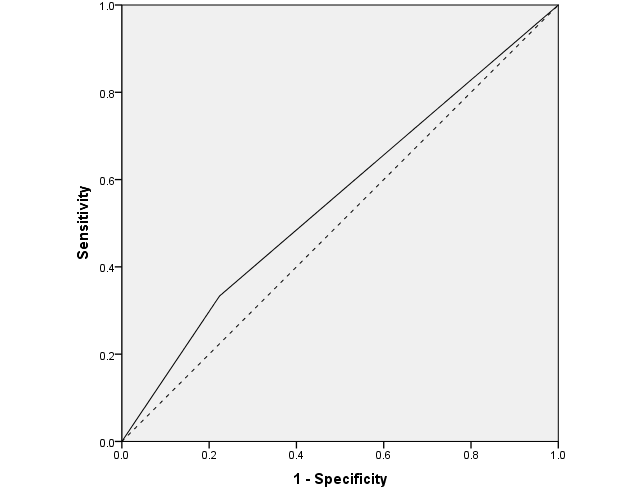


Sensitivity = 4 / 12 = 0.3333 = 33.33%

Specificity = 52 / 67 = 0.7761 = 77.61%

Positive Predictive Value = 4 / 19 = 0.2105 = 21.05%

Negative Predictive Value = 52 / 60 = 0.8667 = 86.67%

| Area Under the Curve (Ultrasound vascular invasion) | | | | |
| --- | --- | --- | --- | --- |
| Area | Std. Error | P-value | 95% Confidence Interval | |
|  |  |  | Lower Bound | Upper Bound |
| 0.555 | 0.094 | 0.548 | 0.371 | 0.738 |

The area under the ROC curve (0.555) exceeds the area under the 45-degree line (0.5) indicating that the ultrasound vascular invasion has some predictive power. However, the p-value (0.548) exceeds the 0.05 level of significance indicating that this area is not significantly larger than 0.5. This is mainly attributed to the fact that the number of false positives (15) is quite large.

**Ultrasound heterogeneous features**

|  | | Post-operation histology | | Total |
| --- | --- | --- | --- | --- |
|  |  | Non-Benign | Benign |  |
| Ultrasound heterogeneous features | Morphological heterogeneity (Non-Benign) | 1 | 5 | 6 |
|  | No morphological heterogeneity (Benign) | 12 | 62 | 74 |
| Total | | 13 | 67 | 80 |


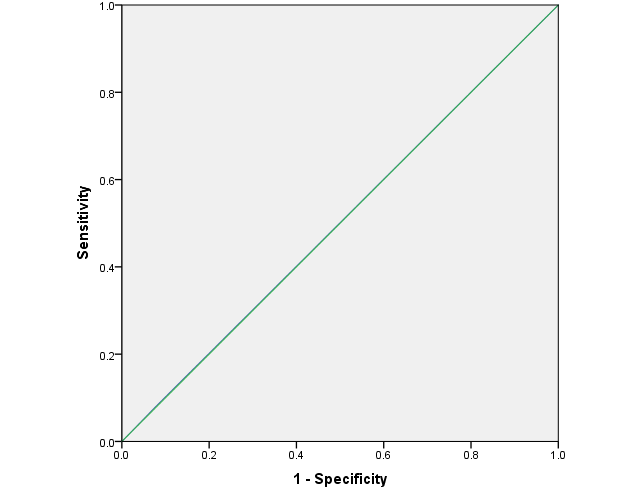


Sensitivity = 1 / 13 = 0.0769 = 7.69%

Specificity = 62 / 67 = 0.9254 = 92.54%

Positive Predictive Value = 1 / 6 = 0.1667 = 16.67%

Negative Predictive Value = 62 / 74 = 0.8373 = 83.73%

| Area Under the Curve (Ultrasound heterogeneous features) | | | | |
| --- | --- | --- | --- | --- |
| Area | Std. Error | P-value | 95% Confidence Interval | |
|  |  |  | Lower Bound | Upper Bound |
| 0.501 | 0.088 | 0.990 | 0.328 | 0.674 |

The area under the ROC curve (0.501) exceeds the area under the 45-degree line (0.5) by a very small margin indicating that the ultrasound heterogeneous feature has very little predictive power. Moreover, the p-value (0.990) exceeds the 0.05 level of significance indicating that this area is not significantly larger than 0.5. This is mainly attributed to the fact that the number of false negatives (12) is quite large.

**Ultrasound septae**

|  | | Post-operation histology | | Total |
| --- | --- | --- | --- | --- |
|  |  | Non-benign | Benign |  |
| Ultrasound septae | Septae present (Non-benign) | 2 | 4 | 6 |
|  | No septae (Benign) | 11 | 63 | 74 |
| Total | | 13 | 67 | 80 |


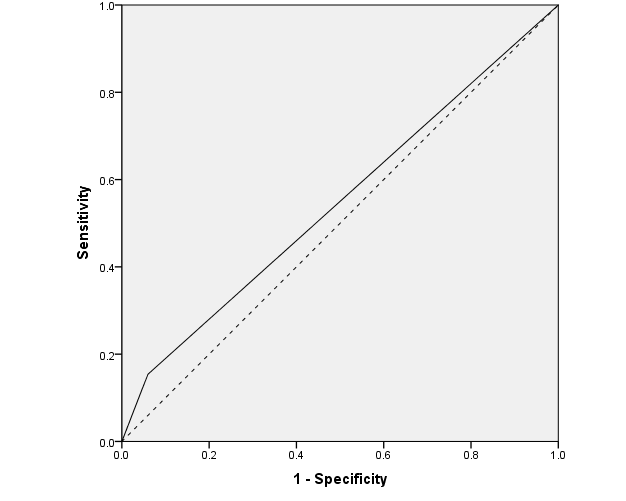


Sensitivity = 2 / 13 = 0.1538 = 15.38%

Specificity = 63 / 67 = 0.9403 = 94.03%

Positive Predictive Value = 2 / 6 = 0.3333 = 33.33%

Negative Predictive Value = 63 / 74 = 0.8514 = 85.14%

| Area Under the Curve (Ultrasound septae) | | | | |
| --- | --- | --- | --- | --- |
| Area | Std. Error | P-value | 95% Confidence Interval | |
|  |  |  | Lower Bound | Upper Bound |
| 0.547 | 0.092 | 0.593 | 0.367 | 0.727 |

The area under the ROC curve (0.547) exceeds the area under the 45-degree line (0.5) indicating that the ultrasound septae has some predictive power. However, the p-value (0.593) exceeds the 0.05 level of significance indicating that this area is not significantly larger than 0.5. This is mainly attributed to the fact that the number of false negatives (11) is quite large.

**Binary Logistic Regression Analysis (Ultra sonographic Tests)**

Logistic regression analysis was used to investigate the collective contribution of Ultrasound depth, ultrasound vascular invasion and ultrasound septae in predicting the outcome (non-benign, benign) of the post-operation histology. This three-predictor logistic regression model explains 10.5% of the post-operation histology outcome (Nagelkerke Pseudo R-Square = 0.105). Moreover, ultrasound depth is a significant predictor because its p-value (0.044) is less than the 0.05 level of significance. So ultrasound depth is the better of the three, followed by ultrasound vascular invasion and ultrasound septae.

The second table displays the odds ratios. The odds ratios all indicate that tumours of size 5cm or more, tumours which include fascia or deep to fascia and previous lipoma in the same position increase the risk that the post-operation histology yields a non-benign tumour since these odds are all larger than 1. However, these odds ratios are not significantly larger than 1 because the p-values exceed the 0.05 level of significance.

| Effect | Model Fitting Criteria | Likelihood Ratio Tests | | |
| --- | --- | --- | --- | --- |
|  | -2 Log Likelihood of Reduced Model | Chi-Square | df | P-value |
| Intercept | 13.544 | 0.000 | 0 | . |
| Ultrasound depth | 17.601 | 4.058 | 1 | 0.044 |
| Ultrasound vascular invasion | 14.989 | 1.445 | 1 | 0.229 |
| Ultrasound septae | 13.618 | 0.074 | 1 | 0.786 |

| Parameter Estimates | | | | | | | |
| --- | --- | --- | --- | --- | --- | --- | --- |
| Post-operation histology | | B | Std. Error | Wald | df | P-value | Odds ratio |
|  | Intercept | -2.749 | 1.367 | 4.043 | 1 | 0.044 |  |
|  | U.S. depth = Fascia/Sub fascial | 1.401 | 0.696 | 4.050 | 1 | 0.044 | 4.059 |
|  | U.S. depth = Superficial | 0 | . | . | 0 | . | . |
|  | U.S. vascular invasion = Yes | 0.905 | 0.739 | 1.500 | 1 | 0.221 | 2.471 |
|  | U.S. vascular invasion = No | 0 | . | . | 0 | . | . |
|  | Ultrasound septae = Present | 0.332 | 1.250 | 0.070 | 1 | 0.791 | 1.394 |
|  | Ultrasound septae = Absent | 0 | . | . | 0 | . | . |

If ultrasound depth is fascial or sub-fascial, the odds that post-operation histology yields a non-benign tumour is 4.059 times when ultrasound depth is superficial.

If the tumour invades microvasculature, the odds that post-operation histology yields a non-benign tumour is 2.471 times when tumour does not invade microvasculature.

If ultrasound septae is present, the odds that post-operation histology yields a non-benign tumour is 1.394 times when septae is not present.

**MRI Size**

|  | | Post-operation histology | | Total |
| --- | --- | --- | --- | --- |
|  |  | Non-Benign | Benign |  |
| MRI Size | 5cm or more on MRI (Non-benign) | 6 | 37 | 43 |
|  | Less than 5cm on MRI (Benign) | 3 | 4 | 7 |
| Total | | 9 | 41 | 50 |


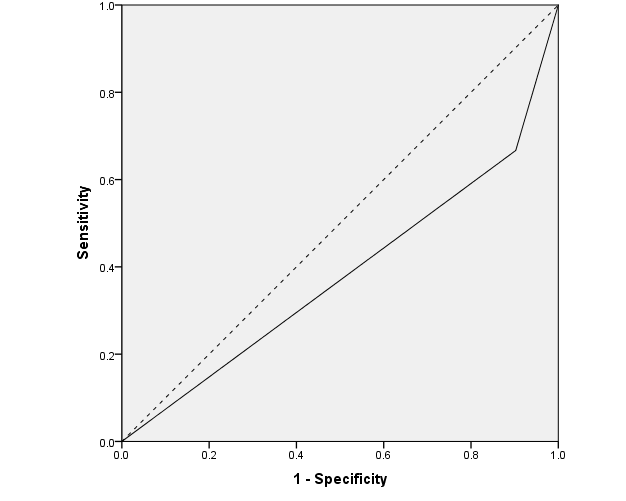


Sensitivity = 6 / 9 = 0.6667 = 66.67%

Specificity = 4 / 41 = 0.0976 = 9.76%

Positive Predictive Value = 6 / 43 = 0.1395 = 13.95%

Negative Predictive Value = 4 / 7 = 0.5714 = 57.14%

| Area Under the Curve (MRI size) | | | | |
| --- | --- | --- | --- | --- |
| Area | Std. Error | P-value | 95% Confidence Interval | |
|  |  |  | Lower Bound | Upper Bound |
| 0.382 | 0.113 | 0.272 | 0.160 | 0.604 |

The area under the ROC curve (0.382) is less than the area under the 45-degree line (0.5) indicating that MRI size has no predictive power. This is mainly attributed to the fact that the number of false positives (37) is large.

**MRI Depth**

|  | | Post-operation histology | | Total |
| --- | --- | --- | --- | --- |
|  |  | Non-Benign | Benign |  |
| MRI Depth | Involves fascia or invades deep to fascia (Non-benign) | 5 | 18 | 23 |
|  | Superficial to fascia on MRI (Benign) | 4 | 23 | 27 |
| Total | | 9 | 41 | 50 |


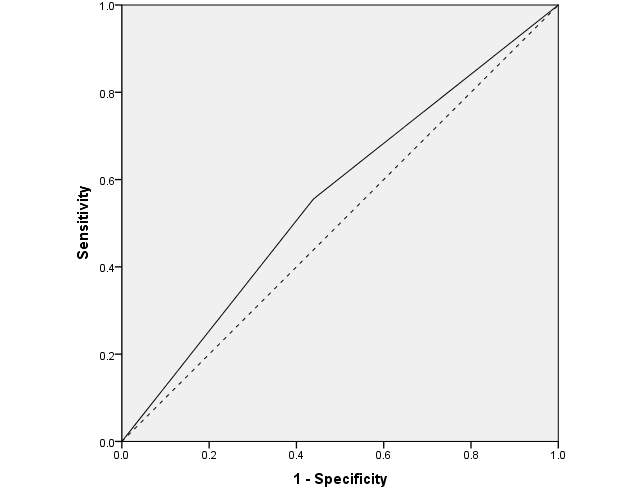


Sensitivity = 5 / 9 = 0.5556 = 55.56%

Specificity = 23 / 41 = 0.5610 = 56.10%

Positive Predictive Value = 5 / 23 = 0.2174 = 21.74%

Negative Predictive Value = 23 / 27 = 0.8519 = 85.19%

| Area Under the Curve (MRI depth) | | | | |
| --- | --- | --- | --- | --- |
| Area | Std. Error | P-value | 95% Confidence Interval | |
|  |  |  | Lower Bound | Upper Bound |
| 0.558 | 0.107 | 0.587 | 0.349 | 0.767 |

The area under the ROC curve (0.558) exceeds the area under the 45-degree line (0.5) indicating that the MRI depth has some predictive power. However, the p-value (0.587) exceeds the 0.05 level of significance indicating that this area is not significantly larger than 0.5. This is mainly attributed to the fact that the number of false positives (18) is quite large.

**MRI Vascular Invasion**

|  | | Post-operation histology | | Total |
| --- | --- | --- | --- | --- |
|  |  | Non-Benign | Benign |  |
| Ultrasound vascular invasion | Invades microvasculature on MRI (Non-Benign) | 0 | 6 | 6 |
|  | Does not invade microvasculature on MRI (Benign) | 9 | 35 | 44 |
| Total | | 9 | 41 | 50 |


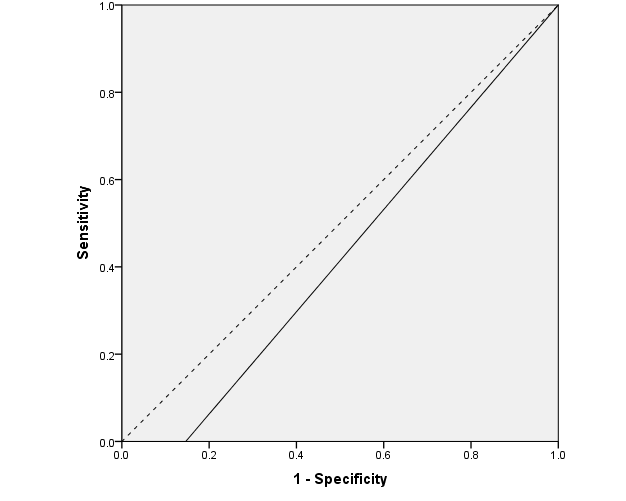


Sensitivity = 0 / 9 = 0.0000 = 0.00%

Specificity = 35 / 41 = 0.8536 = 85.36%

Positive Predictive Value = 0 / 6 = 0.0000 = 0.00%

Negative Predictive Value = 35 / 44 = 0.7955 = 79.55%

| Area Under the Curve (MRI vascular invasion) | | | | |
| --- | --- | --- | --- | --- |
| Area | Std. Error | P-value | 95% Confidence Interval | |
|  |  |  | Lower Bound | Upper Bound |
| 0.427 | 0.097 | 0.495 | 0.236 | 0.618 |

The area under the ROC curve (0.427) is less than the area under the 45-degree line (0.5) indicating that MRI vascular invasion has no predictive power. This is mainly attributed to the fact that the number of false negatives (9) is large.

**MRI heterogeneous features**

|  | | Post-operation histology | | Total |
| --- | --- | --- | --- | --- |
|  |  | Non-Benign | Benign |  |
| MRI heterogeneous features | Heterogeneous (Non-Benign) | 3 | 7 | 10 |
|  | Homogeneous (Benign) | 6 | 34 | 40 |
| Total | | 9 | 41 | 50 |


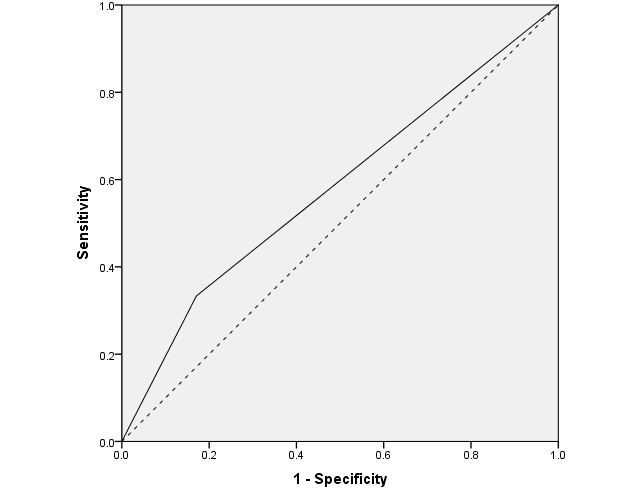


Sensitivity = 3 / 9 = 0.3333 = 33.33%

Specificity = 34 / 41 = 0.8536 = 82.93%

Positive Predictive Value = 3 / 10 = 0.3000 = 30.00%

Negative Predictive Value = 34 / 40 = 0.8500 = 85.00%

| Area Under the Curve (MRI heterogeneous features) | | | | |
| --- | --- | --- | --- | --- |
| Area | Std. Error | P-value | 95% Confidence Interval | |
|  |  |  | Lower Bound | Upper Bound |
| 0.581 | 0.111 | 0.449 | 0.363 | 0.799 |

The area under the ROC curve (0.581) exceeds the area under the 45-degree line (0.5) indicating that the MRI heterogeneous features have some predictive power. However, the p-value (0.449) exceeds the 0.05 level of significance indicating that this area is not significantly larger than 0.5). This is mainly attributed to the fact that the number of false negatives (6) is quite large.

**MRI septae**

|  | | Post-operation histology | | Total |
| --- | --- | --- | --- | --- |
|  |  | Non-benign | Benign |  |
| MRI septae | Septae present (Non-benign) | 1 | 12 | 13 |
|  | Septae absent (Benign) | 8 | 29 | 37 |
| Total | | 9 | 41 | 50 |


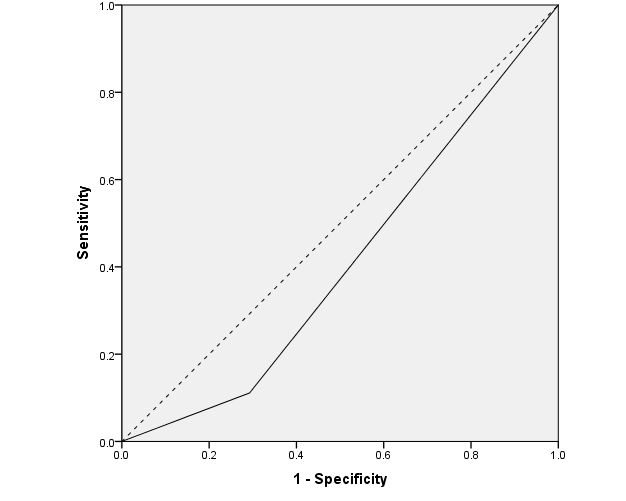


Sensitivity = 1 / 9 = 0.1111 = 11.11%

Specificity = 29 / 41 = 0.7073 = 70.73%

Positive Predictive Value = 1 / 13 = 0.0769 = 7.69%

Negative Predictive Value = 29 / 37 = 0.7838 = 78.38%

| Area Under the Curve (MRI septae) | | | | |
| --- | --- | --- | --- | --- |
| Area | Std. Error | P-value | 95% Confidence Interval | |
|  |  |  | Lower Bound | Upper Bound |
| 0.409 | 0.098 | 0.398 | 0.217 | 0.601 |

The area under the ROC curve (0.409) is less than the area under the 45-degree line (0.5) indicating that MRI septae has no predictive power. This is mainly attributed to the fact that the number of false negatives (8) is large.

**Fat completely suppressed on MRI**

|  | | Post-operation histology | | Total |
| --- | --- | --- | --- | --- |
|  |  | Non-benign | Benign |  |
| Fat completely suppressed on MRI | Fat not completely suppressed (Non-benign) | 1 | 18 | 19 |
|  | Fat completely suppressed (Benign) | 8 | 23 | 31 |
| Total | | 9 | 41 | 50 |


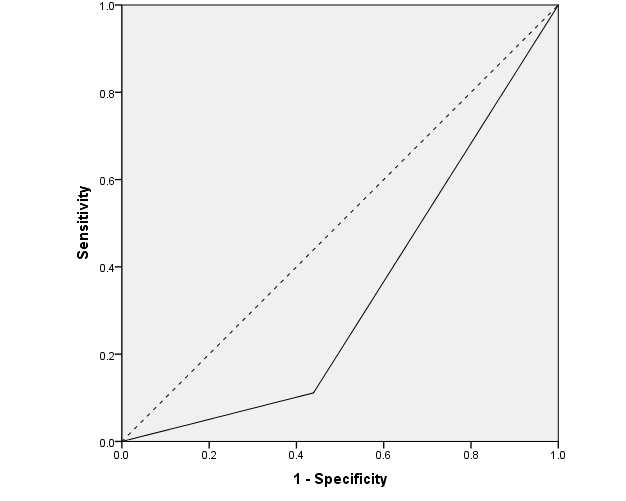


Sensitivity = 1 / 9 = 0.1111 = 11.11%

Specificity = 23 / 41 = 0.5610 = 56.10%

Positive Predictive Value = 1 / 19 = 0.0526 = 5.26%

Negative Predictive Value = 23 / 31 = 0.7419 = 74.19%

| Area Under the Curve (Fat completely suppressed) | | | | |
| --- | --- | --- | --- | --- |
| Area | Std. Error | P-value | 95% Confidence Interval | |
|  |  |  | Lower Bound | Upper Bound |
| 0.336 | 0.090 | 0.127 | 0.159 | 0.513 |

The area under the ROC curve (0.336) is less than the area under the 45-degree line (0.5) indicating that Fat completely suppressed has no predictive power. This is mainly attributed to the fact that the number of false negatives (8) and false positives (18) are large.

**Binary Logistic Regression Analysis (MRI Tests)**

Logistic regression analysis was used to investigate the collective contribution of MRI depth and MRI heterogeneous features in predicting the outcome (non-benign, benign) of the post-operation histology. This two-predictor logistic regression model explains 5.2% of the post-operation histology outcome (Nagelkerke Pseudo R-Square = 0.052). Although none of these two predictors are significant, MRI heterogeneous features is the better of the two, followed by MRI depth.

The second table displays the odds ratios. The odds ratios all indicate that tumours that involve fascia or invades to fascia on MRI and heterogeneous features on MRI increase the risk that the post-operation histology yields a non-benign tumour since these odds are all larger than 1. However, these odds ratios are not significantly larger than 1 because the p-values exceed the 0.05 level of significance.

| Effect | Model Fitting Criteria | Likelihood Ratio Tests | | |
| --- | --- | --- | --- | --- |
|  | -2 Log Likelihood of Reduced Model | Chi-Square | df | P-value |
| Intercept | 10.221 | .000 | 0 | . |
| MRI depth | 10.729 | .507 | 1 | 0.476 |
| MRI heterogeneous features | 11.432 | 1.210 | 1 | 0.271 |

| Parameter Estimates | | | | | | | |
| --- | --- | --- | --- | --- | --- | --- | --- |
| Post-operation histology | | B | Std. Error | Wald | df | P-value | Odds ratio |
|  | Intercept | -2.014 | 0.618 | 10.610 | 1 | 0.001 |  |
|  | MRI depth = Fascia/deep to fascia | 0.535 | 0.756 | 0.502 | 1 | 0.479 | 1.708 |
|  | MRI depth = Superficial to fascia | 0 | . | . | 0 | . | . |
|  | MRI features = Heterogeneous | 0.939 | 0.830 | 1.279 | 1 | 0.258 | 2.557 |
|  | MRI features = Homogeneous | 0 | . | . | 0 | . | . |

If MRI depth involves fascia or reaches deep to fascia, the odds that post-operation histology yields a non-benign tumour is 1.708 times when MRI depth is superficial to fascia.

If MRI features are heterogeneous, the odds that post-operation histology yields a non-benign tumour is 2.557 times when MRI features are homogeneous.

**Binary Logistic Regression Analysis (Clinical and Ultra sonographic Tests combined)**

Logistic regression analysis was used to investigate the collective contribution of size of tumour, depth of tumour, previous Lipoma, ultrasound depth, ultrasound vascular invasion and ultrasound septae in predicting the outcome (non-benign, benign) of the post-operation histology. This six-predictor logistic regression model explains 14.7% of the post-operation histology outcome (Nagelkerke Pseudo R-Square = 0.147). Although none of these six predictors are significant, ultrasound depth is the better of the six, followed by size of tumour, ultrasound vascular invasion, previous lipoma, ultrasound septae and depth of tumour.

| Effect | Model Fitting Criteria | Likelihood Ratio Tests | | |
| --- | --- | --- | --- | --- |
|  | -2 Log Likelihood of Reduced Model | Chi-Square | df | P-value |
| Intercept | 24.766 | 0.000 | 0 | . |
| Size of tumour | 26.449 | 1.684 | 1 | 0.194 |
| Depth of tumour | 24.771 | 0.005 | 1 | 0.944 |
| Previous lipoma | 25.749 | 0.983 | 1 | 0.321 |
| Ultrasound depth | 27.173 | 2.408 | 1 | 0.121 |
| Ultrasound vascular invasion | 26.334 | 1.569 | 1 | 0.210 |
| Ultrasound septae | 24.788 | 0.022 | 1 | 0.881 |

**Conclusions**

- Of the clinical tests, depth of tumour is the best predictor of the post-operation histology outcome (non-benign, benign), followed by size of tumour and previous lipoma.
- Of the ultrasound tests, ultrasound depth is the best predictor of the post-operation histology outcome (non-benign, benign), followed by ultrasound vascular invasion and ultrasound septae.
- Of the MRI tests, heterogeneous features on MRI is the best predictor of the post-operation histology outcome (non-benign, benign), followed by MRI depth.
- Of the clinical and ultrasound tests combined, ultrasound depth is the best predictor of the post-operation histology outcome (non-benign, benign), followed by size of tumour, ultrasound vascular invasion and previous lipoma.
- Ultrasound tests (Pseudo R-Square = 0.105) are more predictive of the post-operation histology outcome than Clinical tests (Pseudo R-Square = 0.082) and MRI tests (Pseudo R-Square = 0.052)
- Ultrasound and Clinical tests combined (Pseudo R-Square = 0.147) are more predictive of the post-operation histology outcome than MRI tests (Pseudo R-Square = 0.052).
